# Genome Sequence of Pluralibacter gergoviae ECO77, a Multireplicon Isolate of Industrial Origin

**DOI:** 10.1128/MRA.01561-19

**Published:** 2020-02-27

**Authors:** Edward Cunningham-Oakes, Tom Pointon, Barry Murphy, Thomas R. Connor, Eshwar Mahenthiralingam

**Affiliations:** a Microbiomes, Microbes, and Informatics Group, Organisms and Environment Division, Cardiff School of Biosciences, Cardiff University, Cardiff, United Kingdom; b Unilever Research and Development, Port Sunlight, Bebbington, United Kingdom; Portland State University

## Abstract

In order to expand the limited understanding of the genomics of antimicrobial-resistant industrial bacteria, we report the genome sequence of Pluralibacter gergoviae ECO77, a historical contaminant strain of industrial origin. The multireplicon 6.16-Mbp genome of ECO77 consists of a 5.37-Mbp main chromosome, a megaplasmid (605,666 bp), and a large plasmid (182,007 bp).

## ANNOUNCEMENT

*Pluralibacter gergoviae* is a member of the *Enterobacteriaceae* family (1) that is occasionally associated with human infection but is commonly associated with cosmetic spoilage (2). In previous studies, we showed that industrial Pseudomonas aeruginosa strains uniquely possess large segments of DNA known as megaplasmids, which confer extra genomic capacity with ≥500 genes and thus may play a role in preservative resistance (3). Furthermore, studies with *Pluralibacter* strains and other Gram-negative bacteria have shown that there are clear links between preservative resistance and outer membrane modifications (4, 5). The genetic changes underpinning these mechanisms of resistance are not well understood but may be elucidated with advances in genomics (6).

Genomic DNA was extracted from pelleted 3-ml overnight cultures of strain ECO77 using a Maxwell 16 tissue DNA purification kit and instrument (Promega) (3). Short-read sequencing was performed with an Illumina NextSeq 500 system, using an NEBNext Ultra II DNA library preparation kit for Illumina (New England Biolabs). Long-read sequencing libraries were produced using a rapid barcoding kit (product number SQK-RBK004; the barcode for ECO77 was BC06) and were sequenced using a MinION R9.4.1 flow cell (Oxford Nanopore Technologies), and bases were called using MinKNOW base-calling software (Oxford Nanopore Technologies). Adaptors were trimmed from short reads using Trim Galore v0.4.4 (7) and from long reads using Porechop v0.2.4 (https://github.com/rrwick/Porechop). Short-read quality was assessed using FastQC v0.11.7 (8), and long-read quality was assessed using Canu v1.8 (9). A hybrid assembly of 6.16 Mbp was generated using Unicycler v0.4.8 (10). Genome quality was assessed using QUAST v5.0.2 (11). All summary statistics are shown in Table 1.

**TABLE 1:** Summary of ECO77 sequencing read and assembly quality metrics

Component	No. of reads (*N*_50_ [bp]) with:		Assembly size (bp)	GC content (%)	*N*_50_ (bp)
	Illumina sequencing (accession no. ERS3910022)	Nanopore sequencing (accession no. ERS3910023)			
Whole genome (accession no. ERS4259183)	2,351,986 (150)	96,499 (62,034)	6,155,741	57.84	5,368,409
Main chromosome			5,368,409	59.27	5,368,409
Megaplasmid			605,666	47.74	294,260
Large plasmid			182,007	53.17	182,007

A total of 5,632 coding DNA sequences were identified in the genome by Prokka v1.14.5 (12). The ECO77 strain also contained 22 rRNAs, 85 tRNAs, and 1 transfer-messenger RNA (tmRNA). The main chromosome was subjected to average nucleotide identity (ANI) analysis against all P. gergoviae NCBI RefSeq genomes using PyANI (https://github.com/widdowquinn/pyani), which confirmed the species-level identity as P. gergoviae.

The plasmid *repA* gene (13) (a highly conserved plasmid gene responsible for copy number), as derived from the Prokka gene annotation of each replicon, was used to identify related homologous plasmids by using BLASTn to align the sequences with the NCBI nucleotide collection. Top hits for the megaplasmid were plasmids from multiple genera within the family *Enterobacteriaceae*, and those for the large plasmid were plasmids from the genus *Klebsiella*.

The contribution of the multireplicon genome of P. gergoviae to preservative tolerance and fitness in industrial settings remains to be determined. Nonetheless, the contribution of the ECO77 genome to public databases provides an invaluable reference genome from an industrial background, where genomic characterization is limited.

### Data availability.

Illumina and Nanopore raw sequence reads and the genome have been deposited in the European Nucleotide Archive (ENA) under ENA project accession number PRJEB34950 and BioSample accession numbers ERS3910022, ERS3910023, and ERS4259183.
